# Calibration of a new transient thermal dissipation–based Internet of Things (IoT)-sensor for xylem sap flow density measurements for three different temperate tree species

**DOI:** 10.1093/treephys/tpaf047

**Published:** 2025-04-16

**Authors:** David Dluhosch, Shahla Asgharinia, Francesco Renzi, Benjamin D Hesse, Lasse Löffelbein, Riccardo Valentini, Thorsten E E Grams, Timo Gebhardt

**Affiliations:** Professorship for Land Surface-Atmosphere Interactions, AG Ecophysiology of Plants, School of Life Sciences, Technical University of Munich (TUM), Hans-Carl-von-Carlowitz Platz 2, 85354 Freising, Germany; Department for Innovation in Biological, Agro-food and Forest Systems (DIBAF), Tuscia University, Via De Lellis, 01100 Viterbo (VT), Italy; Department for Innovation in Biological, Agro-food and Forest Systems (DIBAF), Tuscia University, Via De Lellis, 01100 Viterbo (VT), Italy; Professorship for Land Surface-Atmosphere Interactions, AG Ecophysiology of Plants, School of Life Sciences, Technical University of Munich (TUM), Hans-Carl-von-Carlowitz Platz 2, 85354 Freising, Germany; Department of Ecosystem Management, Climate and Biodiversity, Institute of Botany (BOT), BOKU University, Gregor-Mendel-Straße 33, 1180 Vienna, Austria; Professorship for Land Surface-Atmosphere Interactions, AG Ecophysiology of Plants, School of Life Sciences, Technical University of Munich (TUM), Hans-Carl-von-Carlowitz Platz 2, 85354 Freising, Germany; Department for Innovation in Biological, Agro-food and Forest Systems (DIBAF), Tuscia University, Via De Lellis, 01100 Viterbo (VT), Italy; Professorship for Land Surface-Atmosphere Interactions, AG Ecophysiology of Plants, School of Life Sciences, Technical University of Munich (TUM), Hans-Carl-von-Carlowitz Platz 2, 85354 Freising, Germany; Assistant Professorship of Forest and Agroforest Systems, School of Life Sciences, Technical University of Munich (TUM), Hans-Carl-von-Carlowitz Platz 2, 85354 Freising, Germany

**Keywords:** *Abies alba* Mill. (fir), cooling phase, cyclic regime, *Fagus sylvatica* L. (beech), flow index, heating phase, *Quercus robur* L. (oak), TreeTalker

## Abstract

Measuring xylem sap flow is crucial for calculating water use in trees and forests, but remote measurements are challenging, especially regarding power supply. The transient thermal dissipation (TTD) system addresses these challenges by being power-efficient and robust. This study assesses the TreeTalker© system (version TT+), a newly developed battery-powered, Internet of Things (IoT)-based measurement system with new probes in terms of structure and material compared with previous versions. To calibrate the system, different flow indices (K_i_) were derived for various heating/cooling cycles at several sap flow densities (SFD) using an artificial hydraulic column made of sawdust. To provide a calibration equation for different species, freshly cut stem segments with three distinct xylem structures of temperate trees were used under controlled conditions: *Quercus robur* L. (oak, ring-porous), *Abies alba* Mill. (fir, coniferous) and *Fagus sylvatica* L. (beech, diffuse-porous). A linear model best described the relationship between K_i_ and SFD for sawdust and oak, while a power function suited fir and beech. Compared with the literature, the regression steepness increased with the conduit size. Using the sawdust equation for oak resulted in a 55 ± 3% underestimation of SFD. For fir and beech, using the sawdust equation overestimated daily water use by 149 ± 116% and 71 ± 41%, respectively. A multi-media equation across all tested media reduced the underestimation in oak to 19 ± 3% but increase the overestimation for fir and beech compared with the sawdust equation to 352 ± 210% and 209 ± 75%, respectively. For more accurate estimation of absolute values, species-specific calibration equations are recommended assess SFD using the TTD method. However, there appears to be a dependency when comparing different linear calibration equations with the corresponding conduit sizes of the tree species.

## Introduction

Through higher temperatures and reduced summer precipitation, climate change increases drought stress and negatively impacts water balance of trees and whole forest stands (Hartmann et al. 2018, Schuldt et al. 2020). Consequently, measuring the water balance in trees and stands has become a key research area (Gebhardt et al. 2014, 2023, Brinkmann et al. 2016, Poyatos et al. 2021). Numerous studies have investigated different methodologies and sensors for measuring sap flow density (SFD), as discussed in several recent papers (Smith and Allen 2007, Steppe et al. 2010, Vandegehuchte and Steppe 2013, Köstner et al. 2017, Flo et al. 2019, Poyatos et al. 2021). The IoT-based, battery-powered TreeTalker© (TT+ version 3.2) system was developed to simplify SFD measurement and infrastructure setup for remote measurements. It supports a seamless transition between the thermal dissipation method (TD) and transient thermal dissipation method (TTD) techniques and provides the ability to adjust heating/cooling cycles (H/C cycles) within TTD. Thermal methods have been developed to measure SFD based on changes in sapwood temperature caused by a heat source. The TD method is the most widely used technique for measuring SFD and estimating plant water use/transpiration (Lu et al. 2004, Vandegehuchte and Steppe 2013, Poyatos et al. 2021). The TD method is based on the continuous heating technique developed by Granier (1985, 1987). It consists of two needle-sized sensors—a heater and a reference probe—inserted radially into the sapwood, with the heater probe 10 cm above the reference. Sap flow density in TD is determined using a dimensionless flow index known as Granier K index, K_1_ (Eq. 1), derived from the differential temperature ($\Delta T$) measurements between the heater and reference probes (Granier 1985, 1987). It relies on the zero flow condition to detect daily $\Delta{T}_{max}$ and supports a wide range of positive flow measurements (Lu et al. 2002, 2004) (Table S2 available as Supplementary Data at *Tree Physiology* Online).


(1) \begin{equation*} SFD=119\ast{K_1}^{1.231}\ast \frac{3600}{100000},\kern0.5em where\ {K}_1=\frac{\Delta{T}_{max}-\Delta{T}_i}{\Delta{T}_i} \end{equation*}

Where $\Delta{T}_i$ is the actual temperature difference between the two probes and $\Delta{T}_{max}$ is the maximum temperature difference between the probes measured during a period of zero flow condition. Granier (1985) introduced a universal calibration for TD, but later studies (Steppe et al. 2010, Wullschleger et al. 2011, Sun et al. 2012) emphasized species-specific calibrations for accuracy.

Do and Rocheteau (2002*a*, 2002*b*) developed with the TTD method a discontinuous measurement method based on the TD method. TTD was developed to avoid the influence of natural thermal gradients between the two probes and to obtain a stable zero flux as a reference (Isarangkool Na Ayutthaya et al. 2010). By applying cyclic system (shortening heating duration) the accuracy of TD improves by reducing its sensitivity to nonlinear changes in sapwood temperatures (Isarangkool Na Ayutthaya et al. 2010). Do and Rocheteau (2002b) showed that with a minimum heating time of 10 min at 0.2 W, the system reaches 95% of maximum temperature difference (ΔT_max_), including the advantage of reduced energy consumption compared with the TD-method (Do et al. 2011).

Due to the H/C cycles, it was now also possible to measure with only one sensor (single probe approach) instead of the classical dual probe approach. In the single probe approach the heater probe acts as a singular sensor. The sapwood reference temperature is calculated here with an interpolation of the temperature of the unheated heater probe (Mahjoub et al. 2009, Do et al. 2011, 2018, Masmoudi et al. 2012, Ren et al. 2020).

The TTD method calculates the transient signal ($dT={\varDelta T}_h-{\varDelta T}_c$), the relative temperature change during heating. dT is calculated between the differential cooled temperature ($\Delta{T}_c$, after the cooling phase) and the maximum differential temperature reached after the period of heating ($\Delta{T}_h$). The flow index K_1_, calculated by the same equation as in Granier (1985), includes dT instead of ΔT. A non-species-specific calibration method based on sawdust was employed to convert K_1_ to SFD, as expressed below in Eq. 2 (Do and Rocheteau 2002*a*, 2002*b*).


(2) \begin{equation*} SFD={\left(\frac{11.3\ast{K}_1}{1-{K}_1}\right)}^{0.707},\kern0.5em in\ linear\ form: SFD=10\ast{K}_1 \end{equation*}

Isarangkool Na Ayutthaya et al. (2010) improved TTD calibration by incorporating data from several tropical diffuse-porous trees (Table S2 available as Supplementary Data at *Tree Physiology* Online), proposing a linear relationship: SFD = 12.95 * K_1_. The outcomes exhibited the variability which was found by Do and Rocheteau (2002*b*) in sawdust calibration (Do et al. 2011). Although TTD appears species-independent (Isarangkool Na Ayutthaya et al. 2010, Do et al. 2011), further validation with non-tropical species was recommended. However, notable discrepancies were. To solve the problems observed at high and low flow rates for K_1_  Do et al. (2018) suggested using the flow index K_2_ (Eq. 3). In K_2_ the incremental temperature rise within a window of 30–300 s after the start of the heating calculation is observed. K_2_ was calibrated using sawdust and tropical tree species (Do et al. 2018) (Table S2 available as Supplementary Data at *Tree Physiology* Online).


(3) \begin{equation*} {K}_2=\frac{dT_{max}-{dT}_i}{dT_{max}} \end{equation*}

Mahjoub et al. (2009), applying the theory of heat transfer from the probe to the wood, proposed a different index, I (${t}_i$), using data from the cooling phase. This index includes the transient signal (dT) at both the initial and intermediate times (${t}_i$) of the cooling kinetics. Applying the TTD system to a single probe approach, in the absence of the reference probe, the recorded temperature at the end of the cooling phase where the equilibrium point is considered the stem temperature.


(4) \begin{equation*} I\left({t}_i\right)=\frac{1}{t_i}\mathit{\ln}\ \frac{dT_{t_0}}{dT_{t_i}} \end{equation*}

Where, ${t}_0$ when the heating current is switched off, ${t}_i$ is the intermediate time after kinetics, ${dT}_{t_0}$ is the temperature decrease at the initial time of kinetics and ${dT}_{t_i}$ is the temperature decrease at intermediate time. Using an olive stem segment (*Olea europaea* L.), a calibration curve was assessed to derive the SFD equation in (L dm^−2^ h^−1^) based on index *I* by Mahjoub et al. (2009):


(5) \begin{equation*} SFD\kern0.75em =a\ I\ \left({t}_i\right)+b\kern0.5em \end{equation*}

when


$$ {t}_i=20\ s, $$

*a* = 180 and *b* = − 8.7

The above equation corresponds to the 10/10 cycle as introduced by Do and Rocheteau (2002b) and it is only valid for the positive flow when $I\ \left({t}_i\right)>-\frac{b}{a}$ (Mahjoub et al. 2009). While Mahjoub et al. (2009) tried to eliminate the zero flow dependency, a current weakness of the method is that they did not consider the influence of the conductive heat coefficient, making their calibration coefficient species-specific and thus disregarding the original goal of Granier’s study with K_1_ (Granier 1985). The only solution to eliminate species specificity is to normalize the applied index, as done by Masmoudi et al. (2012). The normalized index K_3_, involving the thermal index under zero flow conditions ${I}_0\ \left({t}_i\right)$, is expressed as:


(6) \begin{equation*} {K}_3=\frac{\ I\ \left({t}_i\right)-{I}_0\ \left({t}_i\right)}{I_0\ \left({t}_i\right)} \end{equation*}

Using data from the olive trunk segment, Masmoudi et al. (2012) proposed a linear regression for SFD estimation (Table S2 available as Supplementary Data at *Tree Physiology* Online). However, Vandegehuchte and Steppe (2012) recommend repeated calibrations in different media to determine if the reported slope is genuinely independent of the medium.

Existing calibration equations of K_1_ for TTD are limited to tropical species and sawdust (Isarangkool Na Ayutthaya et al. 2010, Do et al. 2011). While these studies suggested no need for species-specific calibrations, their applicability to temperate species is uncertain. Lu et al. (2004) showed that changes in sap flow sensor geometry can lead to calibration discrepancies. As differences in TT+ sensors compared with those in earlier studies (Granier 1985, Do and Rocheteau 2002b) may affect calibration (Lu et al. 2004). Furthermore, the TT+ system, applying TTD with different electronical design, remains uncalibrated until this date. This study aims to calibrate SFD measurements using the TT+ system to improve the accuracy of absolute SFD estimates, focusing on:

(i) Comparing the regression accuracy of the flow indices K_1_, K_2_ and K_3_ in sawdust, including an estimation of the impact of different H/C cycles on calibration equations under varying flux rates.
(ii) Comparing species-specific calibration equations to assess SFD using TTD for TT+ with a sawdust and a multi-media equation to derive SFD from K_1_ for different tree species.

## Materials and methods

### T‌T+ sensor and settings

TreeTalker© (TT+ version 3.2) uses a SFD measurement technique classically based on the dual probe system, with a heater probe and a reference probe similar to Granier (1985). The probes were changed in geometry and material compared with Granier (1985). Each probe has a diameter of 0.3 cm and a length of 2 cm, with a thermistor in the center of the needle (Valentini et al. 2019, Matasov et al. 2020, Asgharinia et al. 2022, Niccoli et al. 2023). The heater probe is constructed from copper, optimizing its heating capabilities, while the reference probe is made from fiberglass, integrating a capacitive sensor designed to assess stem water content through the analysis of reference probe data (Asgharinia et al. 2022). The heating probe, designed to consume ⁓0.2 W with a current of 0.06 A and a resistance of 56 Ω, works in tandem with the reference probe to collect the essential temperature data for SFD measurement (Granier 1985). The system offers the TTD method with adaptable settings for H/C cycles, easily adjusted via text message by mobile phone. The default setup of the TT+ provided four temperature gradient data points for reference and heater probe (before heating, at the end of heating, immediately after switching off heating and 20 s into the cooling phase), allowing for the calculation of flow indices K_1_ and K_3_, respectively. The default was 10/50 H/C cycle, i.e., 10 min heating followed by 50 min cooling on an hourly basis. The TT+ software can also be tailored to collect absolute temperature data every second throughout the heat flow curve, enabling the application and comparative evaluation of different flow indices. This setup was essential to capture the heating and cooling phases before and after the heating was turned off for each cycle. Using the TT+ system, to avoid matrix impact on sapwood reference temperature measurements, there is the possibility to use the heater probe data as single probe approach (Do et al. 2011).

### Measurements in an artificial sawdust-filled column

A hydraulic column packed with pine sawdust having a uniform particle size of 2 mm and a dry bulk density of ⁓0.18 g cm^−3^ was used to collect high-frequency heat flow data at various flow rates. The column was 3.2 cm in diameter and 65 cm in length. The experimental setup is based on findings from similar experiments reported by Do and Rocheteau (2002*b*) and Do et al. (2011). Two pairs of TT+ sap flow probes were inserted into a plastic tube 10 cm apart. A water tank was connected above this arrangement to ensure a constant flow through the system. A mini pump maintained the water level in the tank, which automatically refilled the tank when the water level dropped. This setup (Figure S1 available as Supplementary Data at *Tree Physiology* Online) also included a pressure gauge and a digital scale at the base to allow accurate monitoring and adjustment of the flow rates.

### Stem segment preparations

**Figure 1:**
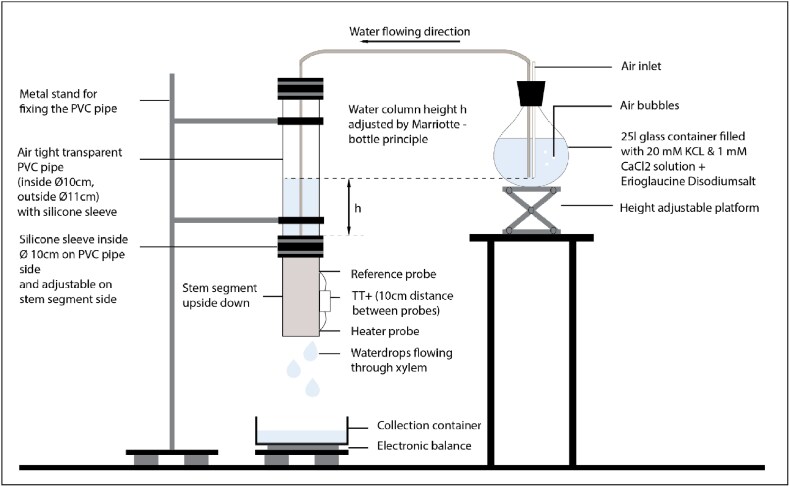
Setup of the Mariotte system in a growth chamber, illustrating the controlled environmental conditions of 25 °C temperature and 75% relative humidity. The variable ℎ represents the height of the water column above the stem segment.

Stem segments of three species *Quercus robur* L. (pedunculate oak, hereafter called oak)*, Abies alba* Mill. (silver fir, hereafter called fir) and *Fagus sylvatica* L. (European beech, hereafter called beech) were used for the calibration. The stem segments were collected from the Kranzberg forest (48 °25′11.3″N 11 °39′23.4″E) near Freising (Bavaria, Germany) with a mean diameter of 8–10 cm. Immediately after cutting, LacBalsam® (COMPO GmbH) was applied to both surfaces to prevent mycosis, and the stem segment was wrapped in plastic foil to prevent evaporation and cavitation during transport to the laboratory. In the laboratory, a branchless segment was truncated before the start of the experiment to a length of 30 cm for beech and fir and 60 cm for oak to account for its greater vessel length (Table 1). The top and bottom of the newly cut stem segment was prepared with a razor blade to open the vessels/tracheids potentially closed by the sawing. On the lower part of the stem segment, 3 cm of bark and cambium was removed to ensure that water would only flow through the xylem. A metal hose clamp attached a silicone sleeve to this part of the segment. The holes for the TT+ sensors were prepared according to the manual (Nature 4.0 TT+ User Manual 2020) (Figure S2 available as Supplementary Data at *Tree Physiology* Online). Subsequently, the entire log with the collar was degassed under water for 24 h using a vacuum pump (ABM Greiffenberger Antriebstechnik GmbH), resulting at a pressure of 0.2 bar to remove potential emboli formed during log preparation. The log segment was then placed upside down in a Mariotte system (McCarthy 1934) (see Supplementary Data) to obtain gravimetric pressure from the water on the underside of the log segment (Steppe et al. 2010) (Figure 1, Figure S1 available as Supplementary Data at *Tree Physiology* Online). A closed 25 L container as a water reservoir was used. This was filled with a 20 mM KCl (Bush et al. 2010) and 1 mM CaCl_2_ (Fuchs et al. 2017) solution. The Mariotte system was used to maintain a constant water column on the underside over a period of time up to 36 h. Water heights in the tube of 30, 20, 10, 5, 2 and 0 cm were measured for 6 h each. Below the stem segment, a water collecting vessel was placed on a balance (model FKB 36 K01-2023e, Kern & Sohn, Max = 36 kg, [d] = 0.1 g) to measure the actual water flow through the xylem of the stem segment every 10 min. After the measurements, the actual water flow on the scale was converted to SFD (L dm^−2^ h^−1^) using the sapwood area, determined by adding Erioglaucine disodium salt (Sigma-Aldrich) to the water solution during measurement in the Mariotte system. The evaporation of water in the collector on the balance was accounted for during the detection of the flow indices under zero flow.

**Table 1:** Parameters of stem segments used in the calibration trial (means ± SD). Mean diameter was calculated from the top and bottom of the segment.

Species	Wood type	Probe length (cm)	N	Mean diameter (cm)	Stem segment length (cm)	Sapwood depth (cm)	Sapwood area (cm^2^)
*Quercus robur* L.	Ring-porous	2	5	9.6 ± 0.9	60.0 ± 0.5	2.2 ± 0.3	49.8 ± 6.8
*Abies alba* Mill.	Coniferous	2	4	8.8 ± 0.9	30.8 ± 1.0	2.9 ± 0.5	58.5 ± 12.7
*Fagus sylvatica* L.	Diffuse-porous	2	4	9.0 ± 1.1	31.3 ± 1.5	2.0 ± 0.3	43.7 ± 5.6

### Calculations of sap flow via balance and sensor data

The default firmware of the TT+ instrument, which collects two temperature data points for each probe over 10/50 H/C cycles, was used to facilitate the calculation of the flow index K_1_ (Eq. 1) and K_3_ (Eq. 6). As ring-porous tree species generally have a relatively steep sapwood profile (Berdanier et al. 2016). The xylem sap flow profile (Berdanier et al. 2016, Gebhardt et al. 2023) was measured to eliminate possible calibration problems with oak as a ring-porous species. The profile was calculated with additional data measured with SFM1 sensors (ICT International, Armidale, NSW 2350, Australia) and the heat ratio method described by Burgess et al. (2001) and Marshall (1958). The SFM1 sensors were inserted into the trunk so that the outer measuring point was at a sapwood depth of 0.3 cm and the inner one was at a depth of 1.8 cm. The correction for the misalignment of the sensor needles from the SFM1 in the trunk was done with the data for zero flow SFD_ref_ (Manual 2014). A linear regression was performed between the measurement point at 1.8 cm and 0.3 cm, to calculate the profile values. The SFD at 1.5 cm on the linear regression of the profile was considered to be representative of 1–2 cm sapwood depth and was calculated as x (Eq. 7). Likewise, SFD at 0.5 cm on the linear regression of the profile was used for the outer 1 cm of sapwood, was estimated to be 100%.


(7) \begin{equation*} x=\frac{SFInner}{SFOuter} \end{equation*}

As in oak the sapwood depth results were consistent with the sensor length (deviation of ±0.3 cm), no adjustments had to be made in the calculation of SFD_ref_. However, the SFD profile showed a decrease of 30% ± 5% in the depth of 1–2 cm compared with the depth of 0–1 cm. The measured profile for oak stem segments was not as steep as reported in other studies with ring-porous species, such as Berdanier et al. (2016). It is assumed that this does not affect the calibration, but only the total flow rate. For fir, no change in SFD with sapwood depth was assumed for young stem segments following Delzon et al. (2004). For beech, no adjustment was required as the sapwood corresponded to the sensor length with a maximum deviation of ±0.3 cm (Table 1).

### Statistics

Analysis and regression for the sawdust and stem segment experiments were carried out using R (version 4.2.2, R Core Team, 2022) in RStudio, using packages such as tidyverse and broom (Wickham et al. 2019). Regression results were presented in two primary forms: linear (Eq. 8) and power (Eq. 9) (following Granier’s classic approach (Granier 1985)). For all regression models, R^2^, *P*-value and RMSE (root mean square error) were calculated to assess the accuracy of the regression (Robinson 2014). In the linear model, the intercept was set to zero and only the slope coefficient a_lin_ was provided to calculate SFD with the flow index K_i_:


(8) \begin{equation*} SFD\ \left(l\ {dm}^{-2}\ {h}^{-1}\right)={a}_{lin}\ast{K}_i \end{equation*}

For the power model, two coefficients, a_power_ and b_power_, were provided to calculate SFD with the flow index K_i_:


(9) \begin{equation*} SFD\ \left(l\ {dm}^{-2}\ {h}^{-1}\right)={a}_{power}\ast{K_i}^{b_{power}} \end{equation*}

R^2^ for power equations was calculated as Pseudo R^2^. Standard errors were estimated for all the coefficients of the given new equations. Statistical differences between the equations were evaluated using an ANOVA. Applying different regression models aims to identify the equation with the highest correlation and accuracy, thereby recommending the most appropriate equation for each specific scenario.

## Results

### Sawdust heat flow curves

**Figure 2:**
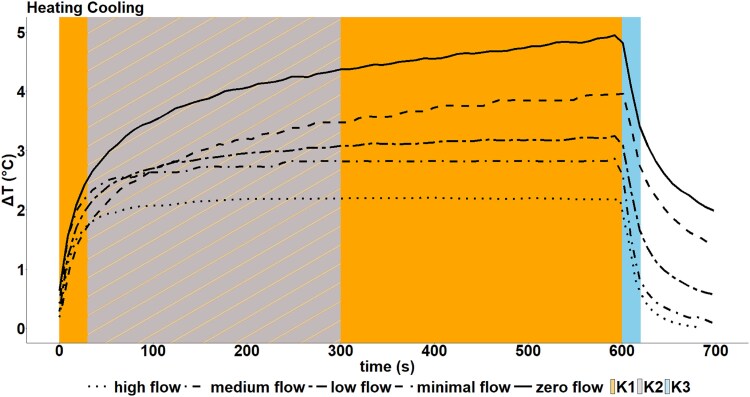
Sample heat flow dynamics in sawdust experiment under the 10/50 H/C cycle: temporal analysis of heating phase, including K_1_ (entire heating phase here 0–600 s, orange), K_2_ (30–300 s in heating phase, grey with orange lines) and cooling phase with K_3_ (0–20 s post current switch-off, blue) under different flow rates. Sap flow density (SFD) is presented as L dm^−2^ h^−1^, in the form of zero flow (0, solid line), minimal flow (0.36, dashed line), low flow (1.7, dotted dashed line), medium flow (4.1, dotted dashed line) and high flow (5.6, dotted line).

With the tailored firmware, high-frequency data were collected to calculate the proposed flow indices: K_1_ (entire heating phase), K_2_ (30–300 s) during the heating phase and K_3_ in the cooling phase, as illustrated in Figure 2. The acquisition of high-frequency data was also crucial for understanding the dynamics of temperature evolution under varying H/C cycles and SFD. During the heating phase, the transient signal did not reach a steady state at minimal flow (SFD = 0.36 L dm^−2^ h^−1^) and zero flow conditions. However, for low, medium and higher flows (e.g., SFD = 1.7, 4.1, 5.6 L dm^−2^ h^−1^), a steady state is achieved within the 600 s.

### Sawdust calibration

**Figure 3:**
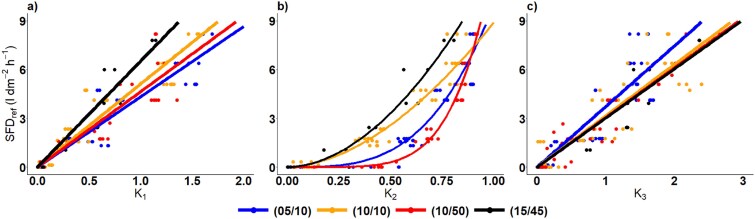
Correlation between the flow indices for four H/C cycles in minutes (5/10 (blue), 10/10 (orange), 10/50 (red) and 15/45 (black)) measured by the TT+ platform and SFD_ref_ measured by the balance, showing the correlation results for (a) K_1_ (entire heating phase), (b) K_2_ (30–300 s in heating) and (c) K_3_ (0–20 s in cooling) in sawdust experiment.

The measured flow indices (K_1_, K_2_, K_3_) and SFD estimated from the balance data were used for the sawdust calibration (Figure 3). For each flow index, four different combinations of H/C cycles (5/10, 10/10, 10/50 and 15/45 min) were used (Figure 3, Table 2). The SFD_ref_ values ranged from 0 to 8 L dm^−2^ h^−1^. K_1_ ranged from 0 to 1.8, K_2_ from 0 to 1 and K_3_ from 0 to 3. The correlation of K_1_ and K_3_ with SFD_ref_ is given in linear form, as the power form did not show any significant improvement in regression accuracy (Table 2). Overall, no significant differences between the different H/C cycles (5/10, 10/10, 10/50, 15/45) in K_1_ were found. However, differences were observed for K_2_ and K_3_, specifically between 10/50 vs 5/10 and between 10/50 vs 10/10 cycles, indicating a variance in regression results depending on the specific heating and cooling durations.

**Table 2:** Comparison of different H/C cycles for flow indices (K_1_, K_2_, K_3_) against reference sap flow density (SFD_ref_) for sawdust experiment. Values a and b are presented with SE.

**Flow index**	**H/C cycle**	**Function-type**	**a**	**b**	***P*-value a**	***P*-value b**	**R** ^**2**^	**RMSE**
**K**_**1**_	5/10	Linear	4.33 ± 0.17	–	<0.001	–	0.85	0.96
	10/10	Linear	5.12 ± 0.16	–	<0.001	–	0.89	0.84
	10/50	Linear	4.65 ± 0.26	–	<0.001	–	0.77	1.15
	15/45	Linear	6.53 ± 0.41	–	<0.001	–	0.90	0.92
**K**_**2**_	5/10	Power	9.97 ± 0.60	3.29 ± 0.29	<0.001	<0.001	0.89	0.86
	10/10	Power	8.89 ± 0.44	1.78 ± 0.14	<0.001	<0.001	0.92	0.73
	10/50	Power	12.73 ± 0.79	5.40 ± 0.37	<0.001	<0.001	0.96	0.50
	15/45	Power	12.23 ± 1.44	1.88 ± 0.27	<0.001	<0.001	0.93	0.76
**K**_**3**_	5/10	Linear	3.73 ± 0.18	–	<0.001	–	0.79	1.24
	10/10	Linear	2.76 ± 0.11	–	<0.001	–	0.82	1.05
	10/50	Linear	3.05 ± 0.09	–	<0.001	–	0.94	0.59
	15/45	Linear	3.03 ± 0.21	–	<0.001	–	0.87	1.02

Interestingly, the results for K_1_ showed that as the heating time increased from 5 min to 10 min, while the cooling time remains constant at 10 min, the slope increases from 4.33 to 5.12 (Figure 3a). This pattern was also observed when the heating time was increased within hourly measurements, from 10 min to 15 min with slopes increasing from 4.65 to 6.53. (Table 2). The R^2^ of 0.77 for the 10/50 cyclic regime is the lowest, while R^2^ for all the other cyclic regimes are ˃0.84.

The correlation between K_2_ and SFD_ref_ is presented as a power function, as the linear form shows significantly lower regression accuracy (Figure 3b and Table 2). In its linear form, the R^2^ value was ˂0.83 and the RMSE ˃1. In contrast, the power form showed improved performance, with an R^2^ value ˃0.89 and an RMSE ˂0.91. The estimated linear slopes for 5/10, 10/10, 10/50 and 15/45 ranged from 4.89 to 8.21. The lowest slope was observed for 10/50 and the highest for 15/45 (Table 2). In the power form, the values for a_power_ varied across the different H/C cycles, ranging from 8.89 to 12.73. The lowest a_power_ value was 8.89, recorded for the 10/10 H/C cycle. In comparison, the ‘b’ values ranged from 1.78 to 5.40.

The results for K_3_, presented in linear form, give slopes for the 5/10, 10/10, 10/50 and 15/45 H/C cycles of 3.73, 2.76, 3.06 and 3.03, respectively, with R^2^ ˃0.79 and RMSE below 1.24 (Figure 3c and Table 2).

### Stem segment calibration

#### Oak

**Figure 4:**
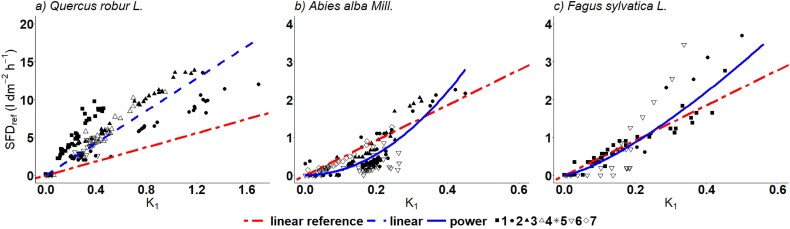
Correlation between the flow index K_1_ and SFD_ref_ (L dm^−2^ h^−1^) with H/C cycle 10/50 and 2 cm probe length for three species: (a) oak, *n* = 5, (b) fir, *n* = 4 and (c) beech, *n* = 4. The shapes of the points show the measured points per stem segment. The dashed-dotted line shows the equation from sawdust (H/C cycle 10/50 and 2 cm probe length) as a linear reference. The dashed line shows the fit of the linear equation. The solid blue shows the fit of the power equation. For each scenario, only the best-fit regression model is presented.

For oak stem segments, SFD_ref_ was in the range of 0–13.87 L dm^−2^ h^−1^ and K_1_ was in the range of 0–1.72. (Figure 4a and Table 3). The correlation between K_1_ and SFD was linear for oak with a significantly higher slope than for the sawdust equation. Using the sawdust equation would lead to a significant underestimation 55 ± 3% (*P* < 0.001) of the SFD compared with the oak-specific function.

**Table 3:** Comparison of regression models (linear and power) for K_1_ with H/C cycle 10/50 and probe length 2 cm measured with TT+ against SFD_ref_ for the three different species (*Quercus robur* L., *Abies alba* Mill. and *Fagus sylvatica* L.), the sawdust calibration and multi-media calibration (three species and sawdust data combined). Values a and b are presented with SE.

**Flow index**	**Species (woodtype)**	**Function-type**	**a**	**b**	***P*-value a**	***P*-value b**	**R** ^**2**^	**RMSE**
**K**_**1**_	*Quercus robur* L. (ring-porous)	Linear	10.44 ± 0.26	–	<0.001	–	0.71	1.95
	*Abies alba* Mill. (coniferous)	Power	14.18 ± 1.44	2.06 ± 0.08	<0.001	<0.001	0.77	0.22
	*Fagus sylvatica* L. (diffuse-porous)	Power	8.75 ± 0.96	1.40 ± 0.10	<0.001	<0.001	0.78	0.47
	multi-media calibration	Linear	8.43 ± 0.19	–	<0.001	–	0.63	1.74
	sawdust calibration	Linear	4.65 ± 0.26	–	<0.001	–	0.77	1.15

#### Fir

For fir, SFD_ref_ was in the range of 0–3 L dm^−2^ h^−1^ and K_1_ of 0–0.47 and, therefore, lower compared with oak (Figure 4b and Table 3). In contrast to sawdust and oak, the relationship between K_1_ and SFD is best described by a power function. Using the sawdust equation would lead to an overestimation of the SFD at K_1_ < 0.35 and an underestimation of SFD at K_1_ > 0.35. Using a linear equation, which is significantly different from the power equation (*P* < 0.001), the relationship for K_1_ and SFD_ref_ showed 68% of the slope of the sawdust equation, even though not significantly different. Compared with the linear equation from oak there is a significant difference. For fir, however, the power model showed a better fit, i.e., higher R^2^, and precision, i.e., lower RMSE, than the linear model.

#### Beech

For beech (Figure 4c and Table 3), SFD_ref_ ranged from 0–4 L dm^−2^ h^−1^ and K_1_ between 0 and 0.6. Like in fir, the power equation shows a better fit between K_1_ and SFD_ref_ with higher R^2^ and precision, i.e., lower RMSE, than for the linear equation. Using the sawdust equation would lead to an underestimation in SFD at K_1_ > 0.35, but not significant though. Using the linear equation, which was significantly different from the power equation (*P* < 0.001), the relationship for K_1_ and SFD_ref_ showed a 1.2 times higher slope compared with the sawdust equation but again not significantly different (Table 3). Furthermore, the equations of beech and fir are not significantly different from each other but are significantly different from the linear equations for oak (*P* < 0.001).

The calculation of K_1_ with the classic dual probe approach was repeated for all three species with the single probe approach. No differences between the two approaches were found (Figure S3 available as Supplementary Data at *Tree Physiology* Online).

#### Multi-media calibration equation

**Figure 5:**
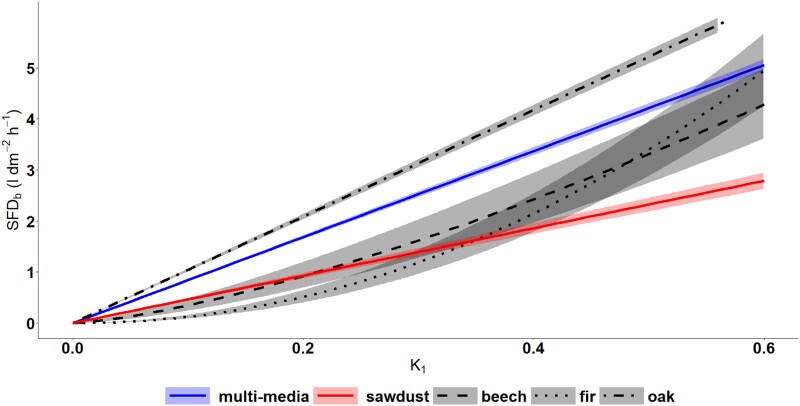
Correlation between the flow index K_1_ and SFD_ref_ (L dm^−2^ h^−1^) with H/C cycle 10/50 for different equations. The equations for oak (dotted-dashed line, *n* = 5), fir (dotted line, *n* = 4) and beech (dashed line, *n* = 4) are compared with the sawdust (solid line, *n* = 5) and the multi-media (solid line, *n* = 18) equations. Only the best-fit regression model with SE (shaded areas) is presented for each equation.

A linear regression equation across all tested matrices, i.e., sawdust and tree species, (Figure 5 and Table 3) shows a nearly doubled slope compared with the sawdust equation. In linear form, the multimedia calibration equation from this study (slope = 8.43) differs significantly from that of Do et al. (2018) (slope = 11.97). This indicates that using the published equation prvided by Do et al. (2018) for the TT+ system would result in an overestimation of SFD by ⁓30%. This multi-media equation would be significantly different from all equations and has a lower R^2^. It reduces the underestimation of oak compared with the sawdust equation. Nevertheless, the underestimation would remain at ⁓19 ± 3%. Furthermore, using the multi-media equation would increase the overestimation of fir and beech compared with the sawdust equation by 81%.

## Discussion

This study determined equations for calculating the SFD from the different measured flow indices (K_1_, K_2_ and K_3_) in different matrices. These equations resulted from the best found correlation between the flow index and SFD_ref_. The correlations were established based on hydraulic bench tests using sawdust compared with correlations for three different tree species. In addition, a multi-media equation across the three species oak, beech and fir and the sawdust was tested.

### Sawdust calibration

Relating the flow indices (K_1_, K_2_ and K_3_) to SFD_ref_ derived with sawdust as the matrix resulted in linear calibration equations for K_1_ and K_3_, whereas a nonlinear, power best-fit function was derived for K_2_. For K_1_, the linear equation resembles that proposed by Granier (1985). This approximation is particularly accurate for the 5/10 and 10/50 H/C cycles, which give almost identical linear slopes with 4.33 and 4.65. However, for the 10/10 and 15/45 cycles, slopes gradually increase to 5.12 and 6.53. The overall average slope across different H/C cycles in this study is 5.16. In contrast Do and Rocheteau (2002*b*) reported a slope of 10.00 for the sawdust matrix, claiming that the calibration equations for K_1_ vs SFD_ref_ remained stable across different H/C cycles. It should be recognized that the artificial hydraulic column setup, size and material of the sap flow probes can also influence the results. Thus, the differences in our study compared with Do and Rocheteau (2002*b*) might be due to the larger size of the probe (3 × 2 mm vs 2 × 2 mm) or the different probe materials (copper vs stainless steel).

To assess the potential impact of hydraulic column diameter on flux turbulence (Do and Rocheteau 2002*b*), the results obtained with a 3.2 cm column were replicated using a 10 cm column. Heat flow curves under zero flux conditions were compared between the two setups, revealing no significant differences and confirming the accuracy of the 3.2 cm column measurements (Figure S3 available as Supplementary Data at *Tree Physiology* Online).

Only a few studies have investigated the relationship between SFD and K_2_ or K_3_ (Mahjoub et al. 2009, Masmoudi et al. 2012, Do et al. 2018, Nhean et al. 2019). However, when our results are compared with those studies, there are apparent differences in the relationship between K_2_ and K_3_ with SFD. For K_2_, our results show a power rather than a linear correlation with SFD, with a dependence on the H/C cycle. However, using the 5/10 H/C cycle, other studies found a linear correlation for K_2_ (Do et al. 2018, Nhean et al. 2019). Interestingly, the equations in these studies differed when comparing different matrices such as sawdust, diffuse-porous and ring-porous wood types.

In the case of K_3_, our sawdust results show the applicability of TTD mainly for medium and high xylem sapflow rates. Comparing our data from the 10/10 H/C cycle with those of Masmoudi et al. (2012), we find clear differences in the slope for the same H/C cycle. For olive trunk segments, an evergreen diffuse-porous species, a slope of 6.10 is reported for the linear correlation between K_3_ and SFD (Masmoudi et al. 2012). Our results for sawdust show approximately half of the reported slope of 2.76 in linear form. Overall, the differences indicate a species/matrix-related difference in the slope of the calibration equations are to be expected. In the heating phase, using K_2_ is recommended to exclude differences cause by probe characteristics, while K_3_ is optimal for high flux rates. In comparison of using K_1_ or K_3_, K_1_ would be the better suited to asses SFD because it can assess low flux rates better than K_3_.

### Calculation of SFD from flow indices is dependent on species

While the TTD approach improves SFD calculations by considering the rapid and nonlinear change in sapwood temperature, it faces a similar challenge as the TD system in detecting zero flow conditions (Do et al. 2011). Detecting zero flow and near-null flows is influenced by the amount and variation of sapwood water content (WC) as extensively investigated in various studies, including Lu et al. (2004), Regalado and Ritter (2007) and Vergeynst et al. (2014). Therefore, the accuracy of the SFD calculation is compromised at SFD < 0.5 L dm^−2^ h^−1^ due to the dependence of the zero flow detection on WC using the heating phase data, as indicated by Do and Rocheteau (2002a), Isarangkool Na Ayutthaya et al. (2010) and Nourtier et al. (2011). With 100–150% WC, coniferous species like fir have a higher WC than oak (WC = 70%) and beech (WC = 80%) (Millers 2013, Ziemińska et al. 2020). In TTD, a minimum heating time of 10 min is known to reach 95% of the maximum temperature difference under zero flow conditions (Do and Rocheteau 2002*b*). However, as shown for fir, this may not be sufficient to achieve steady state conditions. In these cases, the first recommendation would be to increase the heating duration above 10 min to reach steady state. However, using the provided power equations, especially for fir, the problem of zero flow detection is reduced. In terms of regression and accuracy values for K_1_ below 0.05, SFD turns to zero flow.

In contrast to the results of a multi species calibration of tropical trees and sawdust (Isarangkool Na Ayutthaya et al. 2010, Do et al. 2011), our results do not support a multi-media calibration of TTD for temperate tree species and sawdust. Our multi-media calibration equation had a nearly doubled slope than the sawdust equation. However, using the multi-media equation would reduce the underestimation for oak compared with the sawdust equation. In contrast, using the multi-media equation to calculate SFD from K_1_ fir and beech leads to an even higher overestimation than the sawdust equation. Based on our results with sawdust and the multi-media equation, we do not recommend cross-species calibration and suggest species-specific calibrations to asses SFD for temperate tree species, at least for TTD with TT+ sensors. Similar results have been shown by Sun et al. (2012) for TD. However, first results and comparisons with equations from literature indicates a possible dependency of the calibration equation on conduit size for the TTD-method.

### Conduit size affects calibration equation type

For TTD, Do and Rocheteau (2002*b*) recommended a sigmoidal equation to calculate SFD from K_1_ in sawdust. Later Isarangkool Na Ayutthaya et al. (2010) showed a multi-species linear equation for tropical species where the results of Do and Rocheteau (2002*b*) for sawdust fell into the same range (Do et al. 2011). For oak, the linear equation used to calculate SFD from K_1_ is significantly different from the equation used for sawdust. The slope of this equation for oak (slope = 10.44) was similar to Do et al. (2018) (slope = 11.97) and Isarangkool Na Ayutthaya et al. (2010) (slope = 12.95) for tropical diffuse-porous and ring-porous species, respectively. We assume that the xylem vessel diameter of these species to cause this similarity of the slopes as oak has similar large xylem vessels (diameter of 130–420 μm) as the tropical diffuse-porous species *Hevea brasiliensis* ((Willd. ex A.Juss.) Müll.Arg.) and *Mangifera indica* (L.) and tropical ring-porous and *Techtonia grandis* (L.F.) (Richter and Dallwitz 2000, Isarangkool Na Ayutthaya et al. 2010, Do et al. 2018). The only exception is *Citrus maxima* ((Burm.) Merr.), which has small vessel diameters (Sedeek et al. 2017) comparable to fir tracheids.

**Figure 6:**
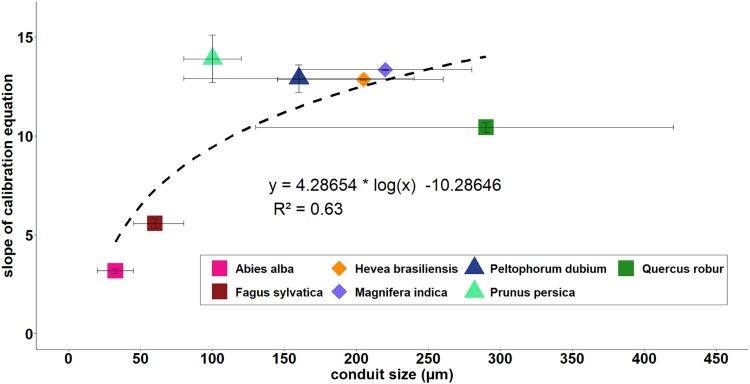
Correlation of the slope of the linear calibration equation (mean ± SE) and conduit size (in μm) for different tree species. The conduit sizes are shown as means with their possible range from literature (Cuny et al. 2014, Hacke and Sauter 1995, Richter and Dallwitz 2000; *WSL xylem database*). Data of the current study is given as squares and data from Paudel et al. (2013) as triangles and Isarangkool Na Ayutthaya et al. (2010) as diamonds. Literature data comprise the tropical diffuse porous wood species *Hevea brasiliensis, Peltophorum dubium* and *Magnifera indica. Prunus persica* and *Quercus robur* are known to form (semi-) ring-porous wood. *Fagus sylvatica* forms diffuse-porous wood and *Abies alba* is a coniferous species. Note the wide range of conduit sizes for oak. The dashed black line represents logarithmic fit.

For beech with slightly smaller vessel diameters (45–80 μm) (Hacke and Sauter 1995, Richter and Dallwitz 2000), a power equation is preferred to calculate SFD from K_1_ for TTD, similar to the results of Fuchs et al. (2017) for TD. Likewise, in fir with a tracheid diameter of 20–45 μm (Cuny et al. 2014), a power equation is advantageous over the conventional linear equation, where the deviation from a possible linear calibration equation is stronger compared with beech. These findings indicates that the equation takes on a power form as the conduit diameter decreases. In addition, comparing our results including literature data suggests a general dependence between conduit size and the slopes of the linear calibration equations, despite differences in sensor design between studies (Figure 6, R^2^ = 0.63). However, the literature review has shown for example in the case of oak that there is a wide range of conduit sizes. This range could be caused by site conditions, such as the formation of smaller tree rings or conduits during drier periods. Because of this relationship, using the provided calibration equations for species with comparable vessel sizes, when no species-specific calibration is available, may reduce the under/overestimation in absolute SFD values. However, there are currently only a limited number of studies on these data and it diviates from the results of Sun et al. (2012) for the TD-method, where species-specific equations are recommended, even for species of the same wood type. Future research should further analyse and refine this relationship.

To show the relevance of the overestimation using the sawdust equation for beech and fir, we calculated the water use of mature trees. As no measurements for fir were available in the field, we took *Pinus sylvestris* L. (Scots pine, further called pine) instead of fir. To our knowledge, there is no calibration equation for pine using TTD, so the power equation from fir was used because pine and fir have similar wood structures. The relevance of the overestimation in beech and pine trees was calculated on a day with an average VPD (Table S1 available as Supplementary Data at *Tree Physiology* Online, Figures S4 and S5 available as Supplementary Data at *Tree Physiology* Online). For comparison, SFD was calculated by K_1_ once with the linear equation from our sawdust results and the recommended power equations. Subsequently, the water use (WU) per tree was calculated with the two SFD values (linear sawdust and power equation) using the model of Berdanier et al. (2016). Compared with the recommended power equation, using the linear sawdust equation would lead to an daily overestimation of 71 ± 41% (mean daily overeastimation 6.38 ± 1.71 L tree^−1^) in WU for beech daily. However, with increasing mean daily VPD, the overestimation of WU is getting reduced in beech trees. For pine, the use of the linear sawdust equation instead of the recommended power equation would lead to an overestimation of 149 ± 116% (mean daily overestimation 16.88 ± 4.29 L tree^−1^) in WU. Using the multi-media calibration equation would lead to an even higher overestimation in WU of pine and beech of 352 ± 210% and 209 ± 75%, respectively. These increased overestimation is caused by the nearly doubled slope compared with the sawdust equation.

The final consideration relates to the feasibility of using heater probe data alone as a single probe approach to calculate the SFD. The regression results are initially derived using data from a dual probe configuration, i.e., heater and reference probes. However, by analysing the temperature data from the heating probe when unheated, interpolation of these temperatures allows the sapwood reference temperature to be estimated. Using this method, regression analyses were repeated for all flow indices, showing negligible differences in the calibration equations between the dual-probe and single-probe approaches (Figure S6 available as Supplementary Data at *Tree Physiology* Online). This underlines the basic possibility of using the provided equations for both approaches. However, the single probe design has the advantages of minimizing tissue damage, speeding up installation and avoiding problems with natural temperature gradients (Do et al. 2011). This modification does not affect the accuracy of the calibration equations for the matrix types analyzed in this study.

## Conclusion

This study highlights the need for different calibration equations to calculate SFD from K_1_, K_2_ and K_3_ using TTD for different matrix types. Here, we provide such equations for three primary categories of wood types: oak (ring-porous), beech (diffuse-porous) and fir (coniferous), including sawdust as a reference and considering the possibility of a multi-media equation. The TTD system is influenced significantly by the matrix type, the detection of the zero-flow state and the amount of heat input. All flow indices show limitations at very low xylem sapflow rates, with K_1_ detecting low flow rates better than K_3_. Sawdust and oak calibration, in our case, showed two different linear equations for calculating SFD from K_1_. However, the accuracy increased for beech and fir using a species-specific power equation. Overall, the outcome clarifies the need for different calibration equations for different species besides the sawdust equation or a multi-media calibration equation.

However, comparing our results with those of other studies indicates that wood properties, such as conduit diameter, may influence the calibration equation. This indicates a possibility of using the provided equations for the three species oak, beech and fir for other species with the according conduit size, where no calibration is available. The use of a multi-media or sawdust calibration equation is not recommended, as it leads to over/underestimations.

Future research should focus on improving the TTD technology as an evolution of the thermal dissipation method valued for its simplicity and widespread use. Enhancements in firmware covering all flow indices, hardware, sensitivity and accuracy should be prioritized while ensuring low cost and power consumption for field applications. In parallel, it is crucial to investigate the effect of wood anatomy, including conduit size and distribution, on sap flow measurements and calibration accuracy. These recommendations aim to contribute to the future development of anatomy-specific calibration equations.

## Supplementary Material

Supplementary_Data_Calibration_of_TTD_major_revision_cleancopy_tpaf047

## Data Availability

Data available on request.
