# Supplementary material for: Calibration of a new transient thermal dissipation–based Internet of Things (IoT)-sensor for xylem sap flow density measurements for three different temperate tree species
Source: Tree Physiol. 2025 Apr 16;45(6):tpaf047. doi: 10.1093/treephys/tpaf047 (PMC12129067; doi:10.1093/treephys/tpaf047)
Supplement: Supplementary_Data_Calibration_of_TTD_major_revision_cleancopy_tpaf047 [file supplementary_data_calibration_of_ttd_major_revision_cleancopy_tpaf047.docx]

# **Supplementary Data**

**Table S1:** Daily water use per tree (WU) calculated using the model of Berdanier et al. (2016) for beech and pine, employing sawdust (WU_sawdust_) and recommended power equations (WU_power_). WU was calculated per tree based on its DBH, with a mean taken across all measured trees (n). Daily mean VPD (VPD_mean_) and Daily max VPD (VPD_max_) are presented in kPA.

| **species** | **n** | **DBH (cm)** | **VPD_mean_ (kPa)** | **VPD_max_ (kPa)** | **WU_sawdust_ (L*tree^-1*^day^-1^)** | **WU_power_ (L*tree^-1*^day^-1^)** |
| --- | --- | --- | --- | --- | --- | --- |
| **beech** | 14 | 37.91 ± 4.26 | 0.70 | 1.41 | 20.25 ± 11.63 | 13.87 ± 10.61 |
| **beech** | 8 | 39.39 ± 2.90 | 1.50 | 2.69 | 30.01 ± 14.31 | 22.33 ± 16.07 |
| **pine** | 16 | 39.51 ± 4.98 | 0.56 | 1.27 | 34.48 ± 11.51 | 17.60 ± 11.03 |

Based on our knowledge and an extensive literature review, mainly on the Transient Thermal Dissipation (TTD) system, Tab. S2 presents calibration results for different species types using different types of flow indices, K_1_, K_2_ and K_3_, incorporating results from the current study. It also includes results from the Thermal Dissipation Probe (TDP) system to ensure a comprehensive analysis. To ensure consistency and ease of comparison, all calibration equations are presented in a standardized linear form.

**Table S2:** Calibration results for different species types using TTD and TDP systems, focusing on TTD, 0.2 watts + classic sensor design

| **Porous media type** | **Tree species** | **Method** | **Flow index** | **Slope (linear)** | **References** |
| --- | --- | --- | --- | --- | --- |
| **coniferous**  **and**  **ring-porous** | *Pseudotsuga menziesii (Mirbel) Franco, Pinus nigra J.F. Arnold,*  *Quercus robur L.* | TDP | K_1_ | 4.30  (Masmoudi et al., 2012) | Granier (1985) |
| **sawdust** | - | TTD (10/10) | K_1_ | 10.00 - 11.00 | Do & Rocheteau (2002b) |
| **tropical diffuse porous** | *H. brasiliensis (Hev.),*  *M. indica (Man.) and*  *C. maxima (Cit.)* | TTD (10/20) | K_1_ | 12.95 | Isarangkool Na Ayutthaya et al. (2010) |
| **tropical diffuse porous + sawdust** | *H. brasiliensis (Hev.),*  *M. indica (Man.) and*  *C. maxima (Cit.)* | TTD (10/10) | K_1_ | 11.97 | Do et al. (2018) |
| **multi species**  **calibration** | *M. domestica*  *P. persica*  *Phelophorum* | TTD (15/15) | K_1_ | 13.45 | Paudel et. al (2013) |
| **tropical diffuse porous + sawdust** | *H. brasiliensis (Hev.),*  *M. indica (Man.) and*  *C. maxima (Cit.)* | TTD (5/10) | K_2_ | 6.42 | Do et al. (2018) |
| **tropical ringporous** | *T. grandis* | TTD (5/10) | K_2_ | 12.78 | Nhean et al. (2019) |
| **tropical diffuse-porous** | *H. brasiliensis,*  *M. indica,*  *E. camaldulensis* | TTD (5/10) | K_2_ | 7.73 | Nhean et al. (2019) |
| **diffuse-porous** | *O. europea* | TTD (10/10) | K_3_ | 6.10 | Masmoudi et al. (2012) |
| **sawdust** | - | TTD (10/10) | K_1_ | 5.12 | current study |
| **sawdust** | - | TTD (5/10) | K_2_ | 5.62 | current study |
| **sawdust** | - | TTD (10/10) | K_3_ | 2.76 | current study |
| **diffuse-porous** | *F. sylvatica* | TTD (10/50) | K_1_ | 5.58 | current study |
| **coniferous** | *A. alba* | TTD (10/50) | K_1_ | 3.18 | current study |
| **ring porous** | *Q. robur* | TTD (10/50) | K_1_ | 10.44 | current study |
| **ring-porous** | *Q. robur* | TTD (10/50) | K_3_ | 9.73 | current study |

For stem segment calibration Teflon tubes were placed inside the closed 25 L container where one tube was used as an air inlet and the other tube led into a plastic tube with the stem piece mounted upside down at the lower end. The water column in the plastic tube (11 cm outer diameter, 10 cm inner diameter) could now be brought to the desired height by the 25 L container, thus creating constant pressure on the underside of the trunk. During the preparation of the Marriotte system, no air reached the underside of the trunk. The 25 L container was connected to the PVC-pipe on the top. A PVC-pipe was connected to the bottom of the wood segment. In the PVC-pipe there is as well a Teflon tube, which is the water inlet in the PVC pipe.


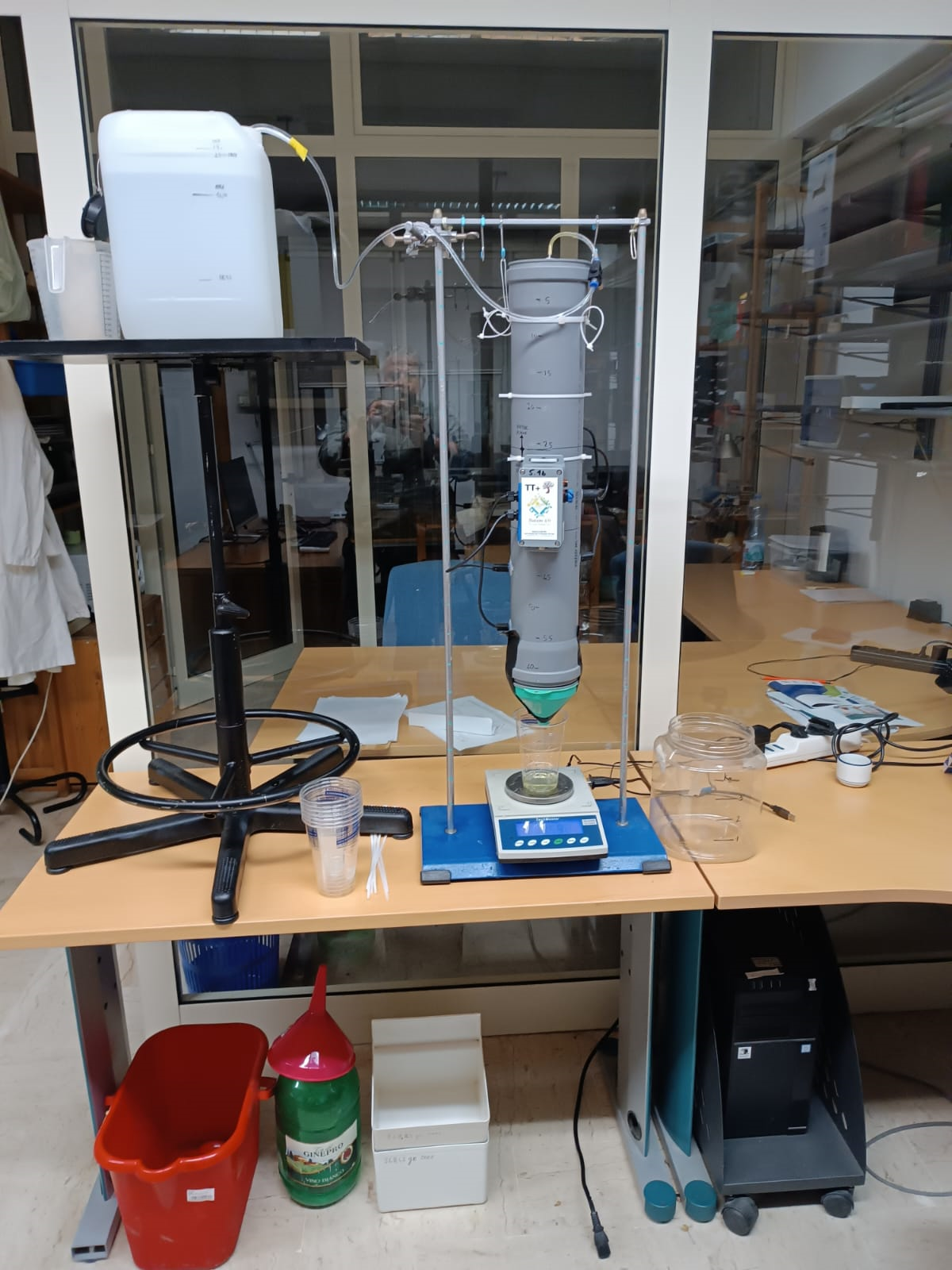

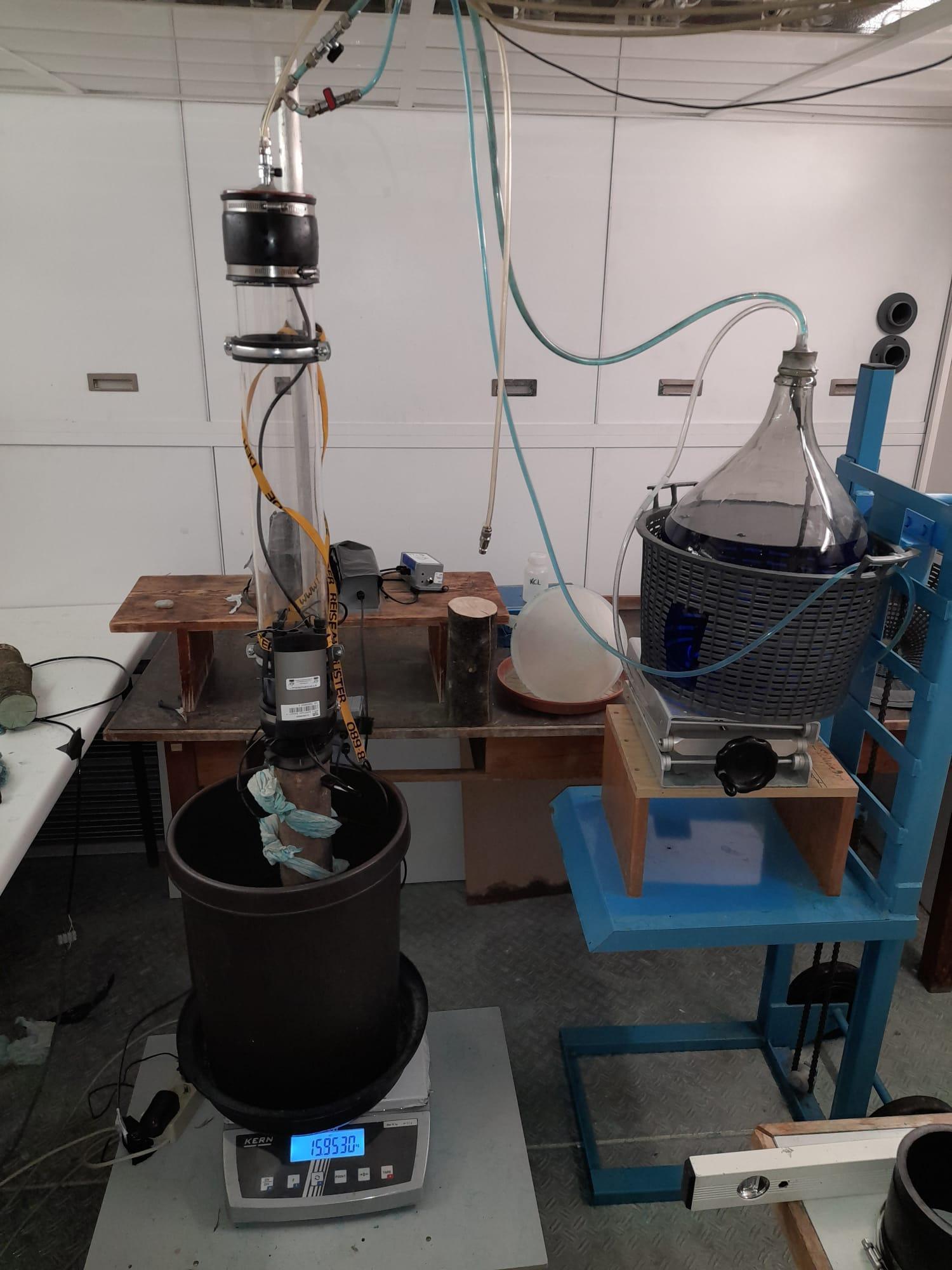


**Figure S1:** Mariotte system setup for sawdust calibration (left picture) and stem segment calibration (right picture).


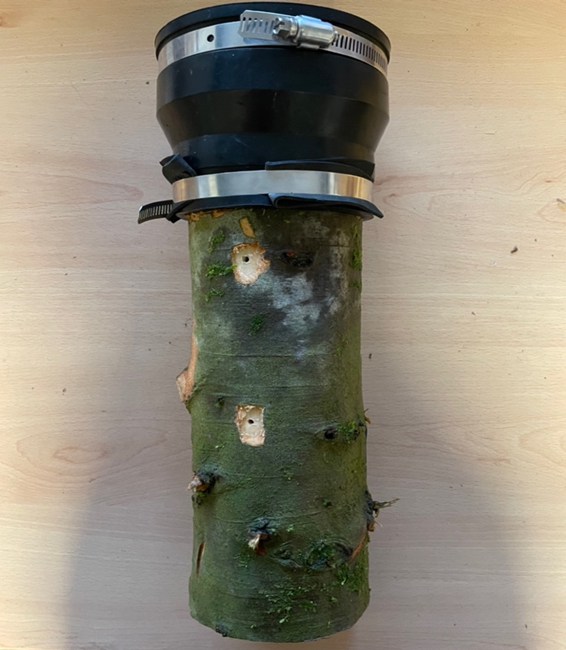


**Figure S2:** Example of a prepared stem segment (beech segment) with silicone sleeve. Both areas where the bark is removed are prepared for the installation of TT+ sensor.


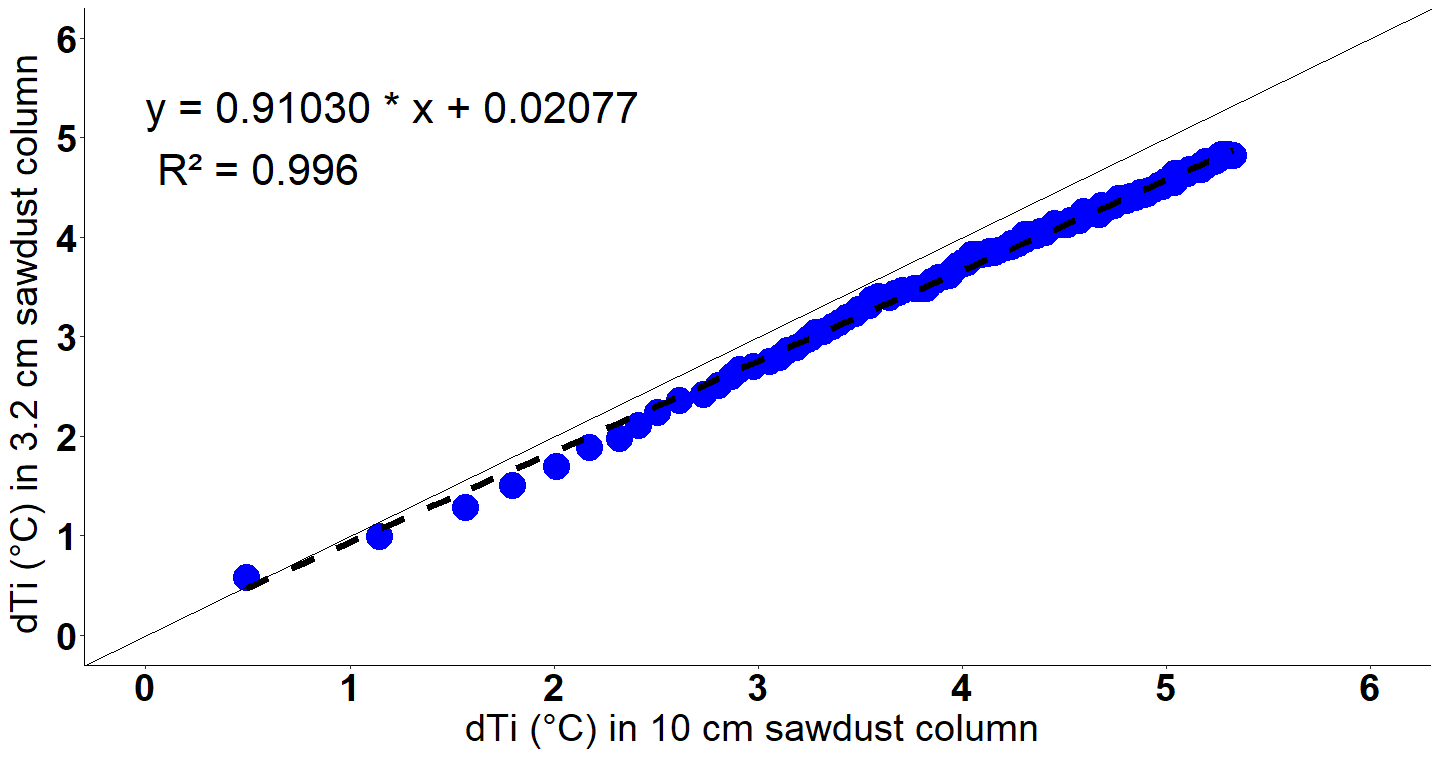


**Figure S3:** Comparison of the measured temperature difference dTi in °C sawdust calibration in a 10 cm and 3.2 cm column in 10/50 H/C cycle. Blue dots show Temperature difference every 3 s during heating phase.


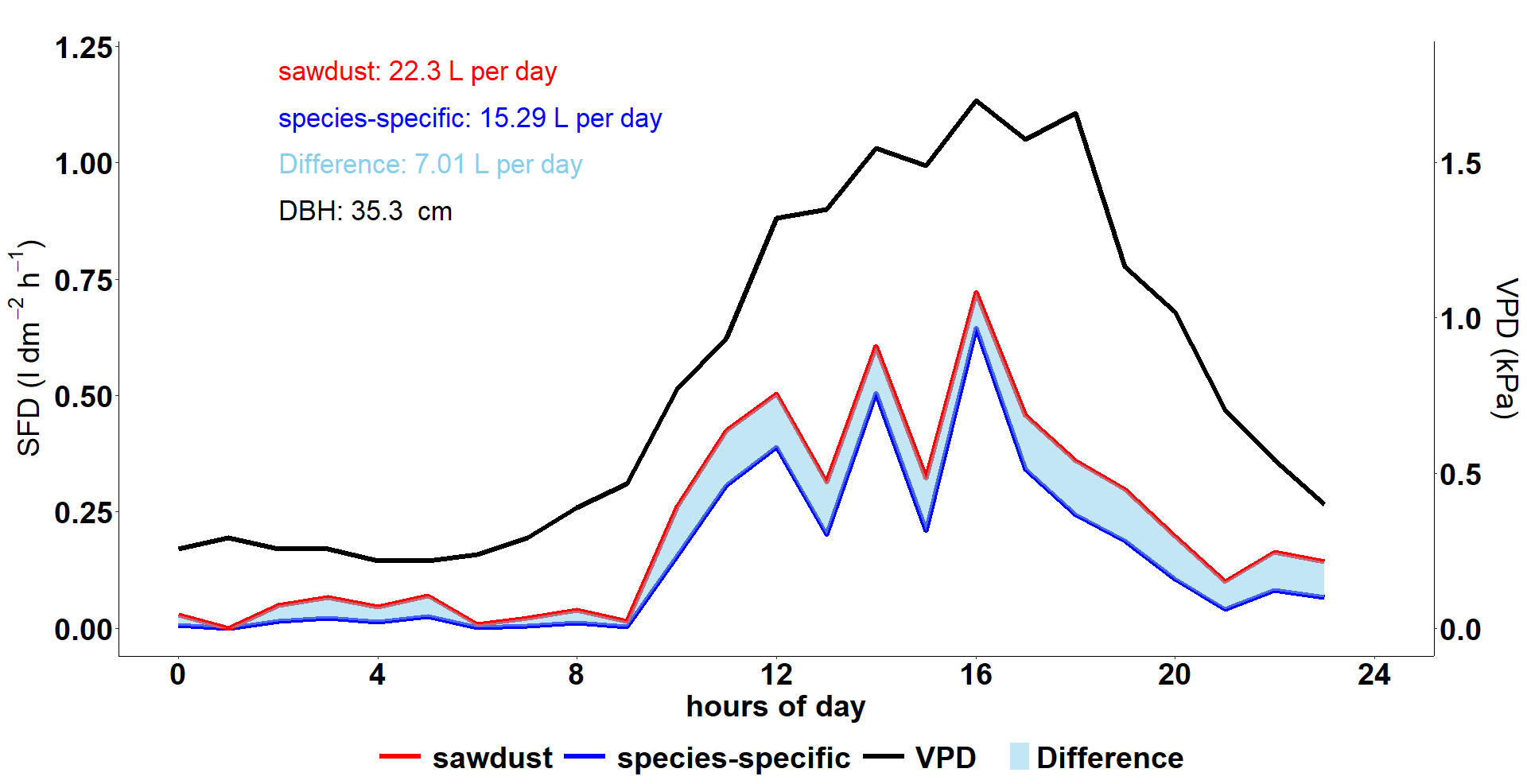


**Figure S4**: Diurnal pattern in SFD (l dm^-2^ h^-1^) comparison of two different equations used for calculation of SFD, linear reference (red) is SFD calculated with sawdust equation, species-specific (blue line) shows SFD calculated with recommended power equation. SFD is calculated for one example beech tree (DBH = 35.3 cm) on one sunny warm day mid of May. VPD (kPa) is included with the black line. The light blue area between the red and the blue line shows the difference between the equations. Calculated water use per day and tree (Berdanier et al., 2016) with sawdust (red) and power (blue) equation and difference are written in the graph.


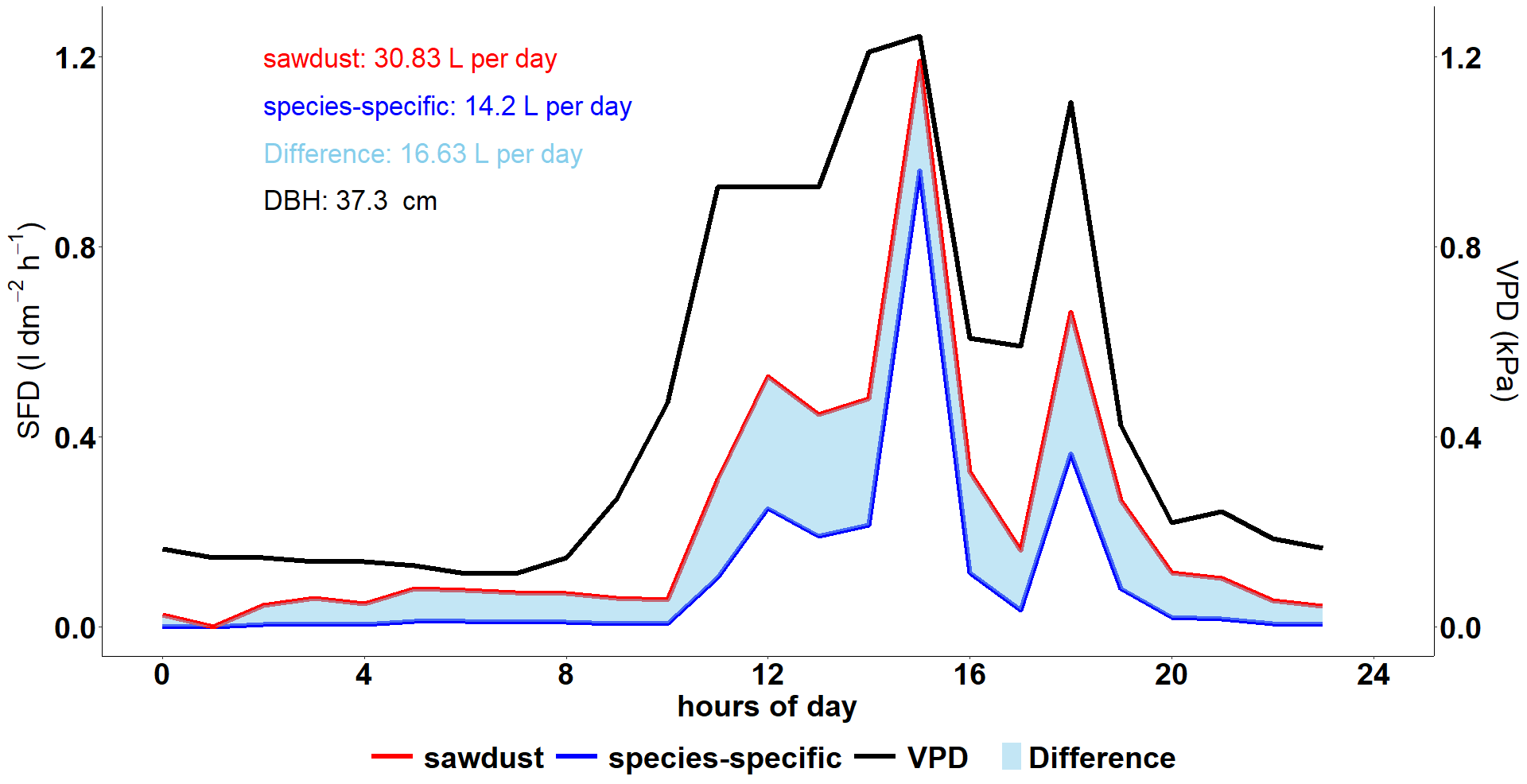


**Figure S5:** Diurnal pattern in SFD (l dm^-2^ h^-1^) comparison of two different equations used for calculation of SFD, linear reference (red) is SFD calculated with sawdust equation, species-specific (blue line) shows SFD calculated with recommended power equation. SFD is calculated for one example pine tree (DBH = 37.3 cm) on one sunny warm day mid of May. VPD (kPa) is included with the black line. The light blue area between the red and the blue line shows the difference between the equations. Calculated water use per day and tree (Berdanier et al., 2016) with sawdust (red) and power (blue) equation and difference are written in the graph.


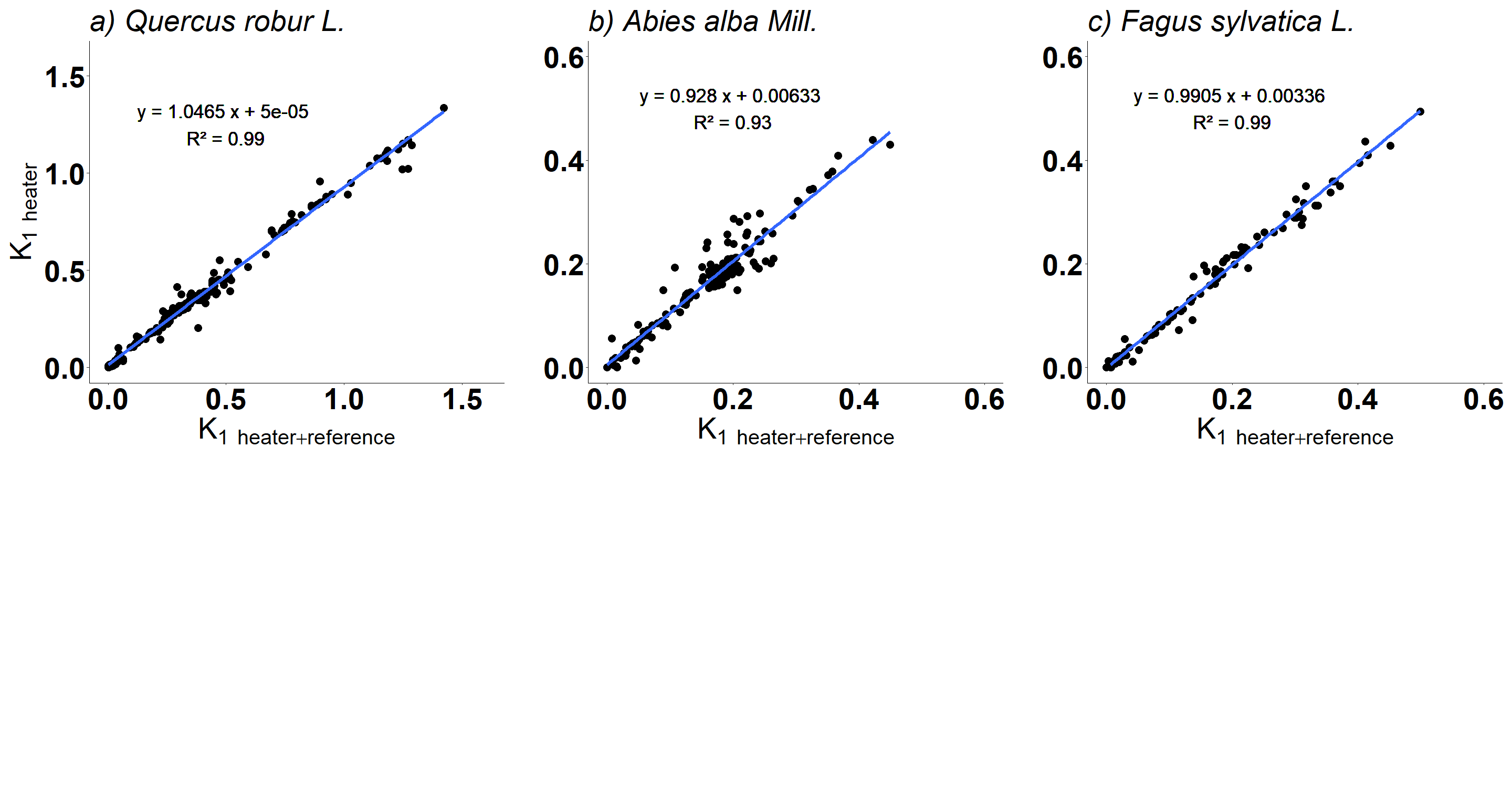


**Figure S6:** K_1_ comparison of Heater and Reference (K_1 heater+reference_) against K_1_ just from heater (K_1 heater_) data from stem segment calibration under constant conditions in climate camber, for a) oak, n = 5 (10/50), 2 cm sensor length, b) fir, n = 4 (10/50), and c) beech, n = 4 (10/50). The points show the measured points from all stem segments. The solid blue line shows the correlation of K_1 heater+reference_ (x) and K_1 heater_ (y) with the equation and R² presented in the graph.
